# The mitochondrial genomes of the acoelomorph worms *Paratomella rubra, Isodiametra pulchra and Archaphanostoma ylvae*

**DOI:** 10.1038/s41598-017-01608-4

**Published:** 2017-05-12

**Authors:** Helen E. Robertson, François Lapraz, Bernhard Egger, Maximilian J. Telford, Philipp H. Schiffer

**Affiliations:** 1 Department of Genetics, Evolution and Environment, University College London, Darwin Building, Gower Street, London, WC1E 6BT UK; 2 CNRS/UMR 7277, institut de Biologie Valrose, iBV, Université de Nice Sophia Antipolis, Parc Valrose, Nice cedex 2, France; 3 Institute of Zoology, University of Innsbruck, Technikerstr. 25, 6020 Innsbruck, Austria

## Abstract

Acoels are small, ubiquitous - but understudied - marine worms with a very simple body plan. Their internal phylogeny is still not fully resolved, and the position of their proposed phylum Xenacoelomorpha remains debated. Here we describe mitochondrial genome sequences from the acoels *Paratomella rubra and Isodiametra pulchra*, and the complete mitochondrial genome of the acoel *Archaphanostoma ylvae*. *The P*. *rubra and A*. *ylvae* sequences are typical for metazoans in size and gene content. The larger *I. pulchra * mitochondrial genome contains both ribosomal genes, 21 tRNAs, but only 11 protein-coding genes. We find evidence suggesting a duplicated sequence in the *I*. *pulchra* mitochondrial genome. The *P*. *rubra, I*. *pulchra and A*. *ylvae* mitochondria have a unique genome organisation in comparison to other metazoan mitochondrial genomes. We found a large degree of protein-coding gene and tRNA overlap with little non-coding sequence in the compact *P*. *rubra* genome. Conversely, the *A*. *ylvae* and *I*. *pulchra* genomes have many long non-coding sequences between genes, likely driving genome size expansion in the latter. Phylogenetic trees inferred from mitochondrial genes retrieve Xenacoelomorpha as an early branching taxon in the deuterostomes. Sequence divergence analysis between *P*. *rubra* sampled in England and Spain indicates cryptic diversity.

## Introduction

Acoel flatworms are small, soft-bodied, unsegmented, marine animals lacking a gut epithelium, coelomic cavity, and anus. Instead, they typically possess a ventral mouth opening, and a simple syncytial digestive system^1^. Due primarily to the common attributes of acoelomate body and the absence of a through gut, Acoela were traditionally grouped as an order within the Platyhelminthes. The first molecular systematic studies on these animals using small subunit (SSU) ribosomal RNA gene sequences revealed that the Acoelomorpha are in fact a distinct lineage, quite separate from the main clade of the Platyhelminthes (Rhabditophora and Catenulida)^2–4^. Instead, these initial molecular studies supported a position of the Acoelomorpha diverging prior to the protostome/deuterostome common ancestor. More recently, the Acoelomorpha have been linked to the similarly simple marine worm *Xenoturbella* in the new phylum Xenacoelomorpha, making sense of their shared simple body plan and other shared morphological characters, such as unusual ciliary ultrastructure^5^ and their simple basiepidermal nervous system^6^. Despite considerable efforts, the position of Xenacoelomorpha within the Metazoa remains unresolved, with alternative lines of evidence placing them either as the sister group to the remaining Bilateria (protostomes and deuterostomes)^7, 8^, or as a phylum within the deuterostomes^9^. A better understanding of acoel phylogeny and evolution is therefore integral to answering central questions concerning the evolution of Bilateria and its subtaxa. To this end more genomic data are needed.

Metazoan mitochondrial DNA (mtDNA) is a closed-circular molecule typically comprising 37 genes which are, for the most part, invariant across the Metazoa^10^. These include the two rRNAs of the mitochondrial ribosome, 22 tRNAs necessary for translation, and 13 protein-coding genes for the enzymes of oxidative phosphorylation. *atp8* is the only gene known to have been commonly lost from this complement, and this has been observed in a number of independent metazoan lineages, including the acoel *Symsagittifera roscoffensis* ^11^. In addition to primary sequence data, mtDNA has a number of other features which can be used for phylogenetic inference, including variations in mitochondrial genetic code^12^; a higher rate of sequence evolution than nuclear DNA^13^; changes in gene order; and changes in the secondary structure of rRNAs and tRNAs^14^.

Mitochondrial gene sequences have been used extensively for phylogenetic inference. In a recent paper, Rouse *et al*. used mitochondrial protein-coding sequence data from four newly discovered species of *Xenoturbella* (*X. hollandorum*, *X. churro*, *X. monstrosa*, and *X. profunda*) to infer the internal phylogeny of the Xenoturbellida^15^. Wider phylogenetic inference including mitochondrial proteins from these species placed Xenacoelomorpha with the deuterostomes^15^, corroborating previous mitochondrial phylogenetic analysis of this phylum^9, 16, 17^.

Mitochondrial gene content is largely invariable across the Metazoa, with the order in which genes are arranged being fairly stable and conserved for up to hundreds of millions of years in some metazoan lineages. Rearrangement events, thought to occur via a model of ‘duplication and deletion’^14, 18^, whereby a portion of the mitochondrial genome is duplicated, and the original copy of the duplicated gene subsequently deleted, are rare. The infrequency of such rearrangements, and the huge number of possible rearrangement scenarios, means that convergence on the same gene order in unrelated lineages is unlikely. Gene order is thus likely to retain evolutionary signals, with a common gene order being indicative of common ancestry and informative for the study of metazoan divergence^19^. Rearrangement of genes within the mitochondrial genome of different species can be a particularly powerful tool in the analysis of phylogenetic relationships^14^ and may also indicate accelerated evolution in a taxon.

In this study, we describe the mitochondrial genomes from three species of acoel: *Paratomella rubra*, *Isodiametra pulchra* and *Archaphanostoma ylvae*. Adult specimens of all animals are approximately 1 mm in length, and, as is typical for small acoel species, they occupy the littoral and sub-littoral zones of marine ecosystems: *P. rubra* has been described across Europe and North America^20, 21^, and *I. pulchra* lives abundantly in the mud flats of Maine^22^. *A. ylvae* has been described from the West coast of Sweden^23^. All species move freely within the sediment by gliding on a multiciliated epidermis. First described by Rieger and Ott^21^, *P. rubra* is an elongate and flattened worm belonging to the family Paratomellidae^24, 25^. A 9.7 kb fragment of mitochondrial genome has previously been described from specimens of *P. rubra* collected on the Mediterranean coast of Spain^26^. *I pulchra* belongs to the family Isodiametridae; it can be maintained long-term in culture and has been used experimentally for *in situ* hybridisation, RNAi, and other molecular protocols^22, 27, 28^. It’s use as a ‘model acoel’ therefore makes this species particularly valuable for investigation. *A. ylvae* also belongs to the Isodiametridae family of acoels. Originally described by Kånneby *et al*. in 2014, its *cox1* gene has been sequenced and used for classification, but no further genes from its mitochondrial genome have been sequenced^23^.

## Results

### Genomic composition

**Figure 1:**
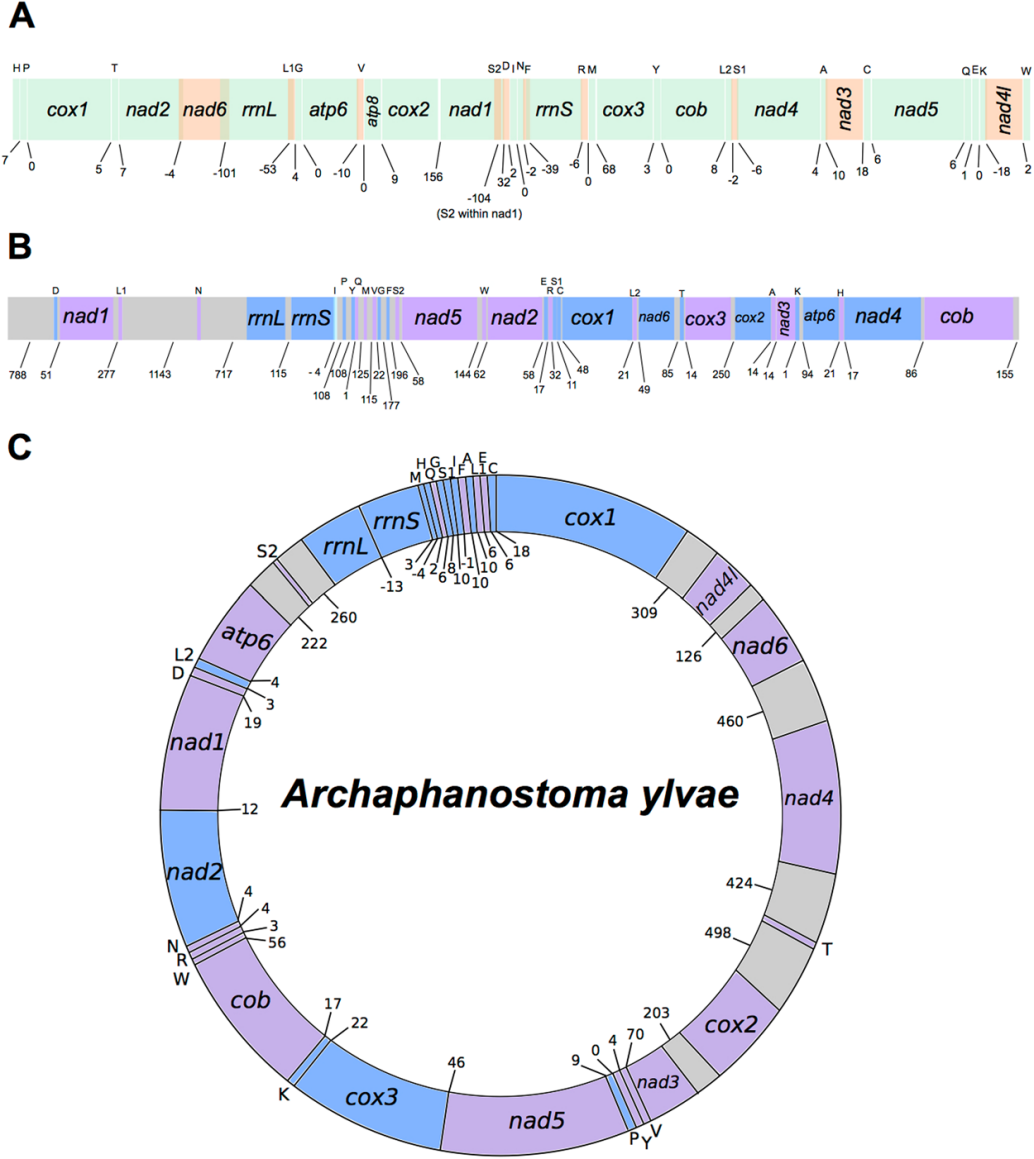
Overview of the mitochondrial genome sequences we resolve for *Paratomella rubra*, *Isodiametra pulchra* and *Archaphanostoma ylvae* (Xenacoelomorpha: Acoela). Genes not drawn to scale. Numbers beneath the sequences show intergenic spaces (positive values) or intergenic overlap (negative values). Protein-coding genes are denoted by three letter abbreviations; ribosomal genes by four letter abbreviations. tRNAs are shown by single uppercase letters. (**A**) *P. rubra* 14,957 base-pair long sequence. All genes found on the positive (forward) strand. Where genes, rRNAs or tRNAs are coloured orange, this is solely to demonstrate overlap with adjacent genes, rRNAs or tRNAs. (**B**) *I. pulchra* 18,725 base-pair long sequence. Genes found on the positive (forward) strand are coloured blue; genes on the negative (reverse) strand are coloured purple. Non-coding sequence shown in grey. (**C**) *A. ylvae* 16,619 nucleotide-long mitochondrial genome. Genes found on the positive (forward) strand are coloured blue; genes on the negative (reverse) strand are coloured purple. Non-coding regions greater than 100 nucleotides in length are shown in grey.

We assembled 14,954 base pairs of the *P. rubra* mitochondrial genome, starting from three genome assembly fragments and using Sanger sequencing of additional PCR fragments (Fig. 1a). We were unable to close the circular mitochondrial genome of *P. rubra*, but our 14.9 kb sequence contains all 13 protein-coding genes, both ribosomal genes and 22 putative tRNAs. Compared to the fragment of the genome previously published we have found four additional protein-coding genes and 12 additional tRNAs^26^. All genes are found exclusively on one strand of the sequence. Allowing for overlap, protein-coding genes account for 74.79% of the genomic sequence; ribosomal genes 13.95%; tRNA genes 9.10%, and non-coding DNA just 2.04%. A 156 nucleotide-long stretch of non-coding sequence is found between *cytochrome c oxidase subunit 2* (*cox2*) and *NADH dehydrogenase subunit 1* (*nad1*).

**Figure 2:**
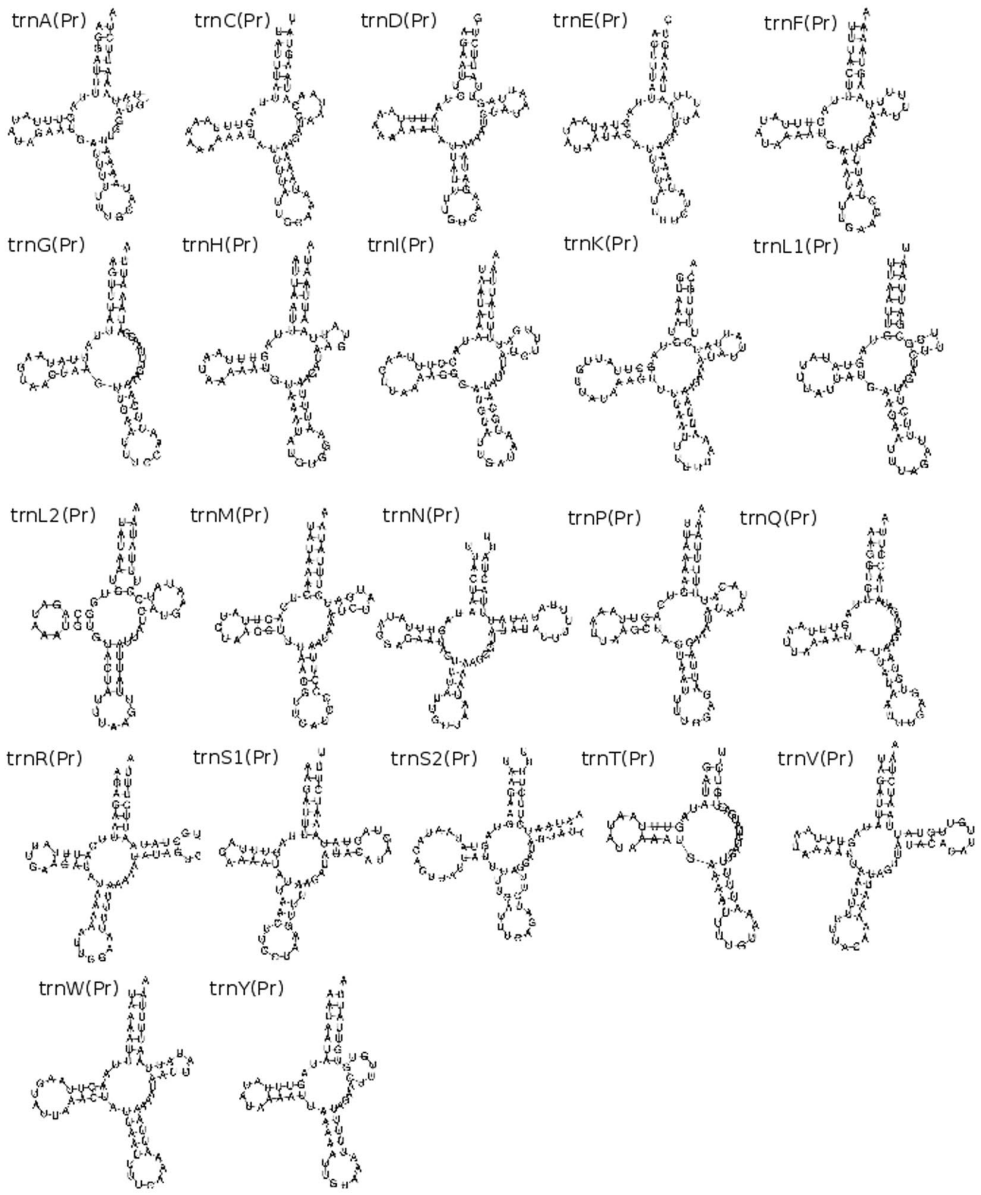
Predicted secondary structures of tRNAs from the mitochondrial genome sequence of *Paratomella rubra* as predicted by MiTFi in Mitos.

In *P. rubra*, *trn*S2 is predicted entirely within the sequence coding for *nad1*, and also has clear deviation from the traditional ‘cloverleaf’ secondary structure of tRNA. In addition, three of the predicted tRNAs have minor overlaps with protein-coding genes: *trn*A with *nad3* (20 nucleotides); *trnK* with *nad4l* (18 nucleotides) and *trnS1* with *nad4* (six nucleotides); and all but five nucleotides of *trnL1* are predicted within the same sequence as *rrnL* (Fig. 1a, Table 1). With the exception of *trnT*, all predicted tRNAs have an amino-acyl acceptor stem composed of seven base pairs, and all predicted tRNAs apart from *trnT* and *trnS2* have a five base pair anticodon stem (Fig. 2). 11 tRNAs have one or two G-T mismatches in their acceptor or anticodon stems (*A*,*C*,*G*,*I*,*K*,*L1*,*L2*,*P*,*Q*,*R*,*T*). All tRNAs have a DHU arm of three or four nucleotides. The structure of the TψC arm shows greater variability, with a number of tRNAs having either a truncated stem, or the arm entirely lacking (Fig. 2).

**Table 1:** Organisation of the Paratomella rubra 14.9 kb mitochondrial genome sequence. All genes found on the ‘positive’ strand.

Feature	Strand	Start	Stop	Length (nucleotides)	Length (AA)	Start Codon	Stop Codon	Intergenic region
*trnH (gtg)*	+	368	426	59				7
*trnP (tgg)*	+	434	495	62				0
*cox1*	+	496	2058	1563	521	ATA	TAA	5
*trnT (tgt)*	+	2064	2123	60				7
*nad2*	+	2131	3105	975	325	ATT	TAG	−4
*nad6*	+	3102	3563	462	154	ATA	TAA	−101
*rrnL*	+	3463	4819	1357				−53
*trnL1 (tag)*	+	4767	4824	58				4
*trnG (tcc)*	+	4829	4887	59				0
*atp6*	+	4888	5496	609	203	ATA	TAG	−10
*trnV (tac)*	+	5487	5553	67				0
*atp8*	+	5554	5730	177	59	ATT	TA-	9
*cox2*	+	5740	6402	663	221	ATT	TAA	156
*nad1*	+	6559	7602	1044	348	ATT	TAA	−104
*trnS2 (tga)*	+	7499	7568	70				32
*trnD (gtc)*	+	7601	7662	62				2
*trnI (gat)*	+	7665	7728	64				0
*trnN (gtt)*	+	7729	7798	70				−2
*trnF (gaa)*	+	7797	7856	60				−39
*rrnS*	+	7818	8547	730				−6
*trnR (tcg)*	+	8542	8608	67				0
*trnM (cat)*	+	8609	8669	61				68
*cox3*	+	8738	9523	786	262	ATT	TAA	3
*trnY (gta)*	+	9527	9585	59				0
*cob*	+	9586	10668	1083	361	ATA	TAA	8
*trnL2 (taa)*	+	10677	10737	61				−2
*trnS1 (gct)*	+	10736	10799	64				−6
*nad4*	+	10794	12119	1326	442	ATC	TAA	4
*trnA (tgc)*	+	12124	12181	58				10
*nad3*	+	12192	12551	360	120	ATT	TAG	18
*trnC (gca)*	+	12570	12629	60				6
*nad5*	+	12636	14387	1752	584	ATA	TAG	6
*trnQ (ttg)*	+	14394	14449	56				1
*trnE (ttc)*	+	14451	14510	60				0
*trnK (ttt)*		14511	14576	66				−18
*nad4l*	+	14559	14867	309	103	ATA	TAA	2
*trnW (tca)*	+	14870	14933	64

**Figure 3:**
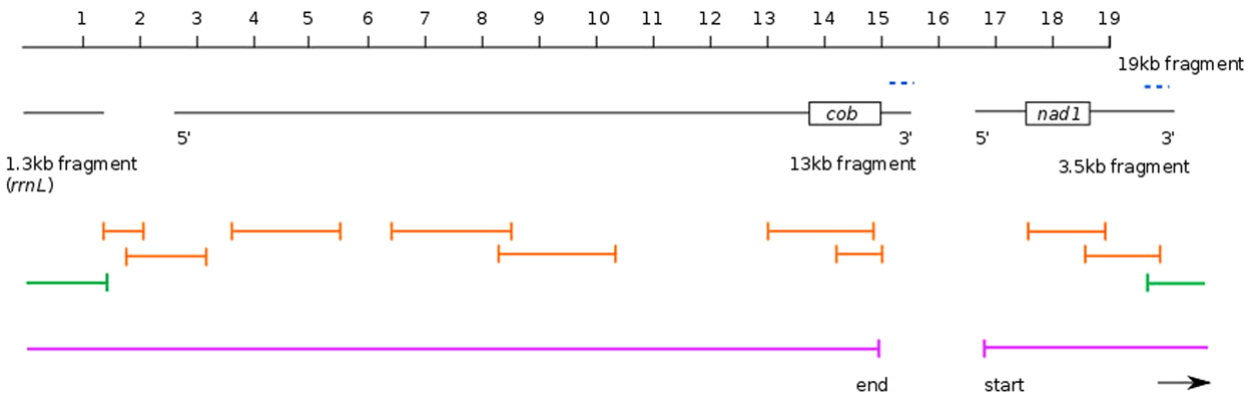
Overview of the initial transcriptome assembly fragments and PCR strategy for scaffolding the *Isodiametra pulchra* mitochondrial genome. 1.3 kb, 13 kb and 3.5 kb fragments aligned to a continuous 19 kb fragment, with the location of the duplicated sequence in the 13 kb and 3.5 kb fragments shown by blue dashed lines. The ‘start’ and ‘end’ regions of the 13 kb and 3.5 kb fragments are annotated by 5′ (start) and 3′ (end). The approximate location of *cob* and *nad1* protein-coding sequence are shown for reference. Reliable PCR-amplicons are shown in orange; the green PCR fragment indicates successful joining of the 3′ end of the 3.5 kb fragment to the *rrnL* fragment, including the duplicated section. The 18,725 base-pair long sequence we resolve is indicated by the pink lines, from ‘start’ to ‘end’.

For *I. pulchra*, we initially recovered a 13 kb contig, a 3.5 kb contig and a 19 kb contig of mitochondrial sequence from our transcriptomic data. The entire 13 kb contig and 2.4 kb of the 3.5 kb contigs were found to be perfectly matching subsets of the longer 19 kb sequence (Fig. 3). We designed several sets of PCR primers to verify the sequence between the 3′ end of the 13 kb and 5′ end of the 3.5 kb fragments found on the long 19 kb fragment (Fig. 3), however, no PCR amplification completely bridged the sequence between the 13 kb to 3.5 kb fragment. We found that the last (3′) 300 bp of the 13 kb fragment was duplicated in the opposite orientation within the end (3′) region of the 3.5 kb fragment. Although the long 19 kb fragment contained the repeated region between the 13 kb and 3.5 kb fragments, we were not able to connect sequences on both sides of the repeated region by PCR. The placement of Sanger sequencing fragments containing the repeat remained ambiguous. We are thus not confident in the assembly of the 19 kb transcriptomic sequence in this section, and therefore treat it as uncertain. Instead we focused on verifying the sequence of the two smaller fragments and on amplifying and sequencing the region lying between them. We reconfirmed the majority of the 13 kb fragment using PCR amplification and Sanger sequencing. We were also able to amplify and sequence fragments joining the 3′ end of the 3.5 kb fragment with the 5′ end of a 1.3 kb fragment containing the *rrnL* gene, which we had identified in transcriptome sequence data using blast. This contig included the duplicated region at the 3′ end of the 3.5 kb fragment (Fig. 3).

In summary, we find the *I. pulchra* mitochondrial genome to have a span of at least 18,725 base pairs (Fig. 1b) based on our PCR validation of the transcriptomic data. This covers the region from the start of the 5′ end of the 3.5 kb sequence which is linked through PCR amplicons to the 5′ end of the 13 kb sequence (including the 1.3 kb *rrnL* contig), and up to the start of the duplicated sequence at the 3′ end of the 13 kb sequence (Fig. 3). As we were not able to bridge the region between the 3′ end of the 13 kb fragment and the 5′ end of the 3.5 kb fragment with PCR, we could not confirm the validity of the duplicated sequence at this position nor fully close the circular mitochondrial genome. It is therefore likely that the entire mitochondrial genome is larger than 19 kb, and may include the duplicated sequence. The sequence we are confident on presenting contains both ribosomal genes, all tRNAs and 11 protein-coding genes. These protein-coding genes and RNAs are encoded on both the plus and minus strands. No sequences resembling either *atp8* or *nad4l* could be found in our sequence.

In the 18.7 kb sequence, protein-coding genes account for 56.66%; ribosomal genes contribute 8.15% and tRNA genes 7.77%. Compared to *the P. rubra* and *S. roscoffensis* mitochondrial genomes, intergenic space in the *I. pulchra* sequences is unusually high: non-coding DNA accounts for 22.72% of the sequences, including 14 intergenic regions of greater than 100 base pairs.

**Figure 4:**
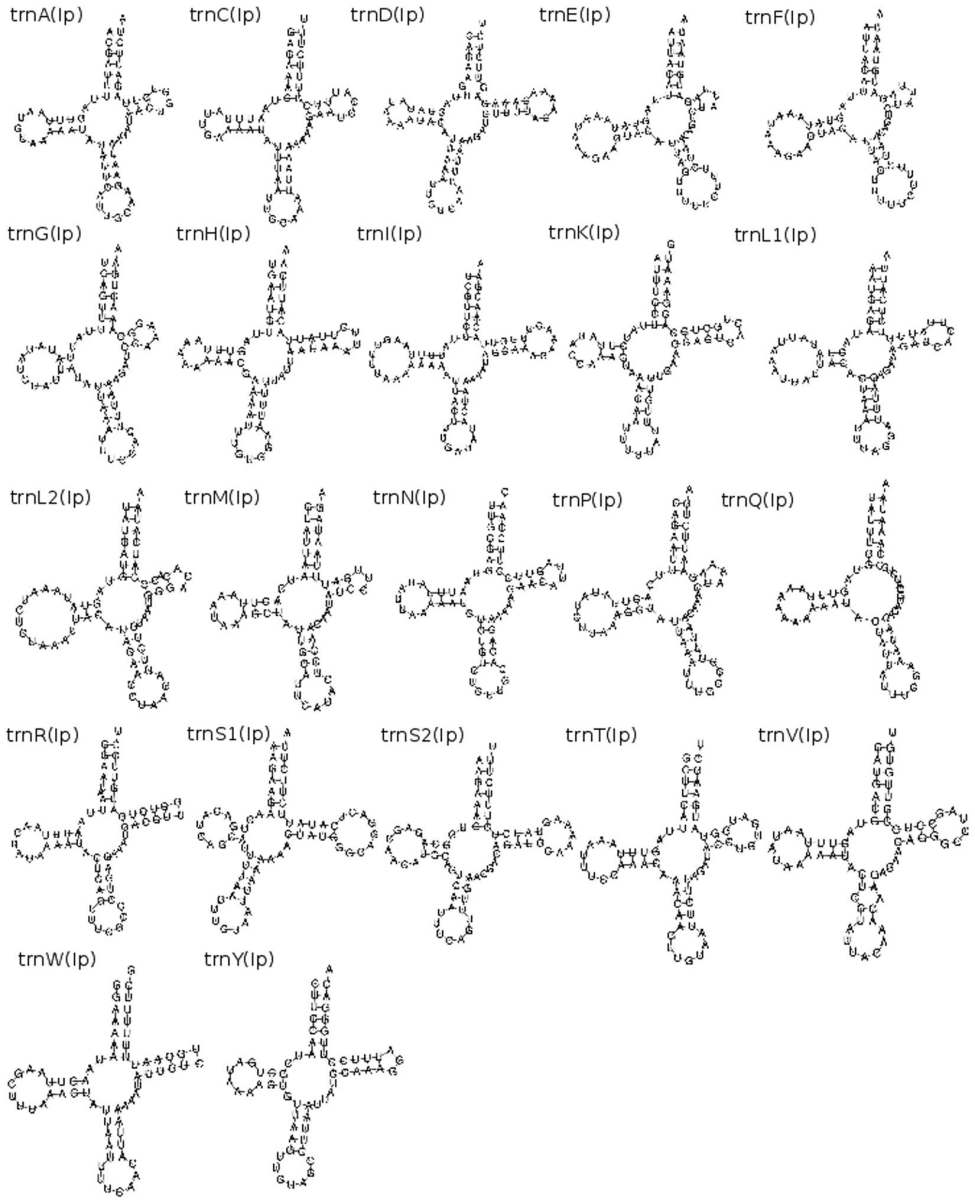
Predicted secondary structure of tRNAs from the mitochondrial genome sequences of and *Isodiametra pulchra* as predicted by MiTFi in Mitos.

We identified all 22 expected tRNAs in the *I. pulchra* mitochondrial genome. Predicted sequences for *rrnS* and *trnI* overlap by four base pairs, but no other overlaps were found between any tRNAs or with any protein-coding genes (Fig. 1b, Table 2). All predicted tRNAs have an amino-acyl acceptor stem composed of seven base pairs and a five base pair anticodon stem, with the exception of *trnE, trnF* and *trnS2*, which have an anticodon stem composed of only four base pairs (Fig. 4). The structure of the DHU arms and TψC show greater variability, and are composed of either 3 or 4, or between 3 and 6, base pairs respectively, across the 22 tRNAs. Whilst the TψC arm is missing entirely in *trnQ*, and very truncated in *trnE*, *trnF*, *trnG* and *trnP*, more of the predicted tRNAs fit the stereotypical ‘cloverleaf’ secondary structure than has been found for other acoel species, including *S. roscoffensis* and *P. rubra* (Fig. 4).

**Table 2:** Organisation of the Isodiametra pulchra 18.7 kb mitochondrial genome sequence.

Feature	Strand	Start	Stop	Length (nucleotides)	Length (AA)	Start Codon	Stop Codon	Intergenic region
*trnD (gtc)*	+	789	848	60				51
*nad1*	−	900	1784	885	295	ATG	TAA	277
*trnL1 (tag)*	−	2062	2130	69				1143
*trnN (gtt)*	−	3274	3339	66				717
*rrnL*	+	4057	4657	601				115
*rrnS*	+	4773	5698	926				−4
*trnI (gat)*	+	5695	5768	74				108
*trnP (tgg)*	+	5877	5939	63				108
*trnY (gta)*	+	6048	6111	64				1
*trnQ (ttg)*	−	6113	6173	61				125
*trnM (cat)*	−	6299	6360	62				115
*trnV (tac)*	−	6476	6543	68				22
*trnG (tcc)*	+	6566	6627	62				177
*trnF (gaa)*	+	6805	6873	69				196
*trnS2 (tga)*	−	7070	7139	70				58
*nad5*	−	7198	8907	1710	570	ATG	TAA	144
*trnW (tca)*	−	9052	9118	67				62
*nad2*	−	9181	10233	1053	351	ATG	TAA	58
*trnE (ttc)*	+	10292	10355	64				17
*trnR (tcg)*	−	10373	10439	67				32
*trnS1 (tct)*	+	10472	10539	68				11
*trnC (gca)*	+	10551	10613	63				48
*cox1*	+	10662	12197	1536	512	ATA	TAG	21
*trnL2 (taa)*	−	12219	12286	68				49
*nad6*	+	12336	12811	476	159	ATG	T−	85
*trnT (tgt)*	+	12897	12963	67				14
*cox3*	−	12978	13775	798	266	ATG	TAA	250
*cox2*	+	14026	14640	615	205	ATA	TAA	14
*trnA (tgc)*	−	14655	14718	64				14
*nad3*	−	14733	15110	378	126	ATG	TAA	1
*trnK (ttt)*	+	15112	15178	67				94
*atp6*	+	15273	15955	683	228	ATA	TA−	21
*trnH (gtg)*	−	15977	16042	66				17
*nad4*	+	16060	17403	1344	448	ATG	TAA	86
*cob*	−	17490	18570	1081	361	ATA	T−	155

The complete, closed circular mitochondrial genome of *A. ylvae* was recovered from genome sequencing data of *P. rubra* specimens collected from Yorkshire, UK. Contamination of the *P. rubra* samples was confirmed using NCBI Blast, which yielded a 99% identical sequence to the published *A. ylvae cox1*. The complete *A. ylvae* mitochondrial genome is 16,619 nucleotides in length, and contains 12 protein-coding genes, both rRNAs, and 22 predicted tRNAs (Fig. 1c, Table 3). With *cox1* at the start of the genome on the ‘positive’ strand, all other protein-coding genes apart from *cox3* and *nad2* are found on the ‘negative’ strand. Both rRNAs are found on the positive strand, and tRNAs are distributed between the two. Accounting for a small amount of overlap between genes - totalling 18 nucleotides of overlap across the whole genome - protein-coding genes make up 64.72% of the genome. tRNAs contribute 8.91%, and rRNAs 9.31%. As found for *I. pulchra*, non-coding DNA makes up a large amount of the genome, totalling 17.17%.

**Table 3:** Organisation of the Archaphanostoma ylvae 16.6 kb mitochondrial genome.

Feature	Strand	Start	Stop	Length (nucleotides)	Length (AA)	Start Codon	Stop Codon	Intergenic
*cox1*	+	1	1539	1539	513	ATA	TAA	309
*nad4l*	−	1849	2133	285	95	ATG	TAA	126
*nad6*	−	2260	2727	468	156	ATG	TAG	460
*nad4*	−	3188	4531	1344	448	ATA	TAA	424
*trnT (tgt)*	−	4956	5025	70				498
*cox2*	−	5524	6171	648	216	ATA	TAA	203
*nad3*	−	6375	6743	369	123	ATG	TAA	70
*trnV (tac)*	−	6814	6882	69				4
*trnY (gta)*	−	6887	6953	67				0
*trnP (tgg)*	+	6954	7022	69				9
*nad5*	−	7032	8687	1656	552	ATG	TAA	46
*cox3*	+	8734	9519	786	262	ATG	TAA	22
*trnK (ttt)*	+	9542	9611	70				17
*cob*	−	9629	10714	1086	362	ATT	TAA	56
*trnW(tca)*	−	10771	10837	67				3
*trnR (tcg)*	−	10841	10908	68				4
*trnN (gtt)*	−	10913	10984	72				4
*nad2*	+	10989	11990	1002	334	ATG	TAA	12
*nad1*	−	12003	12872	870	290	ATG	TAA	19
*trnD (gtc)*	−	12892	12954	63				3
*trnL2 (taa)*	+	12958	13025	68				4
*atp6*	−	13030	13731	702	234	ATA	TAA	222
*trnS2 (tga)*	−	13954	14020	67				260
*rrnL*	+	14281	15089	809				−13
*rrnS*	+	15077	15814	738				3
*trnM (cat)*	+	15818	15879	62				−4
*trnH (gtg)*	+	15876	15945	70				2
*trnQ (ttg)*	−	15948	16007	60				6
*trnG (tcc)*	+	16014	16079	66				8
*trnS1 (tct)*	+	16088	16151	64				10
*trnI (gat)*	+	16162	16231	70				−1
*trnF (gaa)*	−	16231	16296	66				10
*trnA (tgc)*	+	16307	16373	67				10
*trnL1 (tag)*	−	16384	16450	67				6
*trnE (ttc)*	−	16457	16522	66				6
*trnC (gca)*	+	16529	16601	73				18

**Figure 5:**
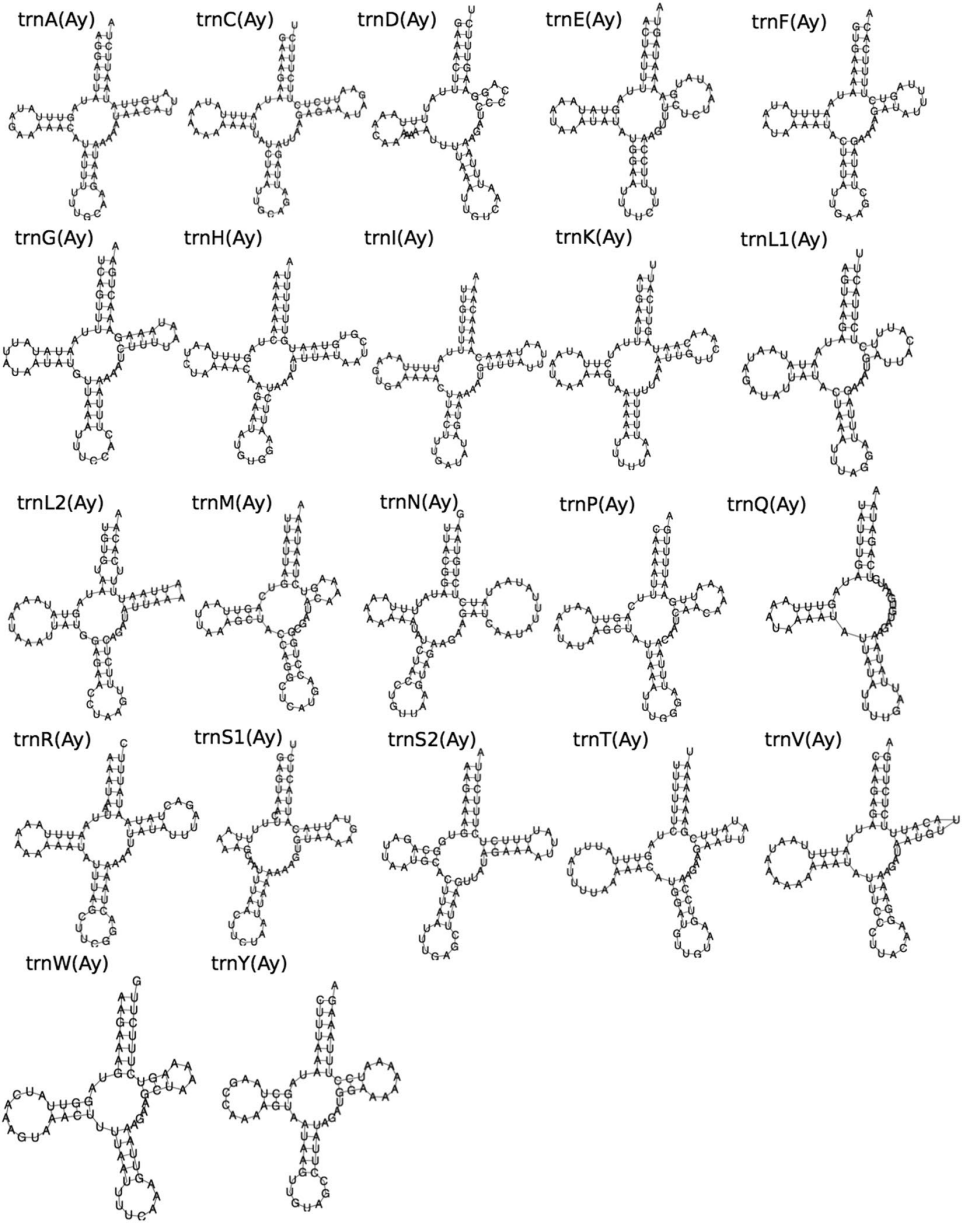
Predicted secondary structure of tRNAs from the mitochondrial genome sequence of *Archaphanostoma ylvae* as predicted by MiTFi in Mitos.

We identified possible sequences for all 22 mitochondrial tRNAs in the *A. ylvae* genome, although four of these (*trnE*, *trnI*, *trnK* and *trnS1*) have an e-value prediction of greater than 0.0001. All predicted secondary structures of the tRNAs in the *A. ylvae* mitochondrial genome have standard-length acceptor and anticodon stems, and the majority – with the exception of *trnK*, *L1*, *L2*, *N*, *S2* and *Y –* have a four nucleotide-long D-loop (Fig. 5). As in the other acoel mitochondrial genomes, the greatest variability is found in the TψC arm, which is truncated in *trnD*, *E*, *F, L1*, *P*, *V*, *W* and *Y* and missing in *trnQ* (Fig. 5).

The *P. rubra* genome is 78.15% A + T rich, which is higher than the A + T content calculated for the *I. pulchra* genomic sequence at 67.28%, and the *A. ylvae* complete genome at 74.70%. Overall nucleotide usage on the plus strand of *P. rubra* is: A = 29.29%; T = 48.86%; C = 6.77% and G = 15.10%; GC-skew = 0.38 and absolute AT-skew = 0.25. Overall nucleotide usage for *I. pulchra* is: A = 34.04%; T = 33.24%; C = 16.45% and G = 16.27%; GC-skew = 0.006 and AT-skew = 0.012. For *A. ylvae*, A = 40.41%; T = 34.29%; C = 12.82% and G = 12.47%; GC-skew = 0.014 and AT-skew = 0.082. GC-skew and AT-skew absolute values for *P. rubra* are much higher than that of *S. roscoffensis*, although the absolute values for *I. pulchra* and *A. ylvae* are comparatively low^11^. AT-skew value for the *P. rubra* sequence is just 0.01 different from that of the published partial *P. rubra* genome, and GC-skew is slightly higher (published *P. rubra* GC-skew = 0.32)^26^.

### Gene order and gene arrangement

All thirteen protein-coding genes in *P. rubra* have complete initiation codons: ATA (x5) and ATT (x8). Five of the protein-coding genes previously published differ in the nucleotide sequence of their start codons: *nad2*, *atp8*, *cox2*, and *cox3* all have ATA as an initiation codon in our analysis, compared to ATT found in previous analysis^26^. Twelve of the genes have full stop codons: TAA (x9) or TAG (x3). *atp8* was found to have a truncated stop codon (TA-), which is thought to be completed during post-transcriptional modification (Table 1). The eleven protein-coding genes found for *I. pulchra* also have full initiation codons: ATA (x4) and ATG (x7). Eight of the genes for this species have full stop codons: TAA (x7) and TAG (x1); *nad6*, *atp6* and *cob* are inferred to have truncated stop codons (Table 2). Initiation codes in *A. ylvae* are: ATA x4, ATG x7 and ATT x1; all genes have TAA as stop codons, with the exception of *nad6*, which has TAG (Table 3). As in other invertebrate mitochondrial genomes, our data indicates a deviation from the ‘standard’ genetic code, with ATA encoding the start codon methionine, M, instead of isoleucine, I.

We found all genes in *P. rubra* on the ‘plus’ strand. In *I. pulchra*, genes are distributed over the plus and minus strands, with just two ‘blocks’ of genes with the same transcriptional polarity clustered together (*rrnL*-*rrnS*-*trnI*-*trnP*-*trnY*; *trnS2*-*nad5*-*trnW*-*nad2*). Similarly, in *A. ylvae* genes are distributed across the two strands, with two clustered ‘blocks’ of genes and tRNAs (*nad4l*-*nad6*-*nad4-trnT-cox2-nad3-trnV-trnY*; *trnM-trnH-trnQ-trnG-trnS1-trnI-trnF-trnA-trnL1-trnE-trnC*). Whilst the *P. rubra* mitochondrial sequence has a large degree of overlap between adjacent genes, the opposite is true for *I. pulchra* and *A. ylvae*. Unlike other metazoan mitochondrial genomes, where genes are adjacent or overlapping and one or two larger non-coding regions are commonly found, *I. pulchra* non-coding sequence is found consistently between protein-coding genes and between tRNAs, ranging in length from eleven to 277 base pairs. In addition, three long non-coding regions of 788, 1143 and 717 base pairs are found at the start of our sequence; between *trnL1* and *trnN*; and *trnN* and *rrnL*. The A + T content of these three sections are 68.78%, 65.79% and 76.15% respectively. The compositional difference between the 717 base pair non-coding region and the rest of the genome is statistically different (χ^2^ = 25.629, p < 0.0001), with a higher A + T content indicating that it could function as a transcriptional control region. The A + T content of the 788 base pair non-coding region is not significantly higher than the rest of the sequence (χ^2^ = 0.85). Similarly, there is a large portion of intergenic, non-coding sequence in the *A. ylvae* genome. Eight regions of non-coding sequence greater than 100 base pairs are found throughout the genome, with 24 additional smaller intergenic regions, ranging in size from 3 to 70 base pairs. Of the larger non-coding sequences, three have an A + T content that is statistically higher than the entire sequence: 309 nucleotides between *cox1* and *nad4l* (χ^2^ = 3.944, p < 0.1); 126 nucleotides between *nad4l* and *nad6* (χ^2^ = 6.964, p < 0.01) and 260 nucleotides between *trnS2* and *rrnL* (χ^2^ = 9.654, p < 0.01). The *P. rubra* sequence has just one longer non-coding intergenic sequence, of 196 base pairs.

**Figure 6:**
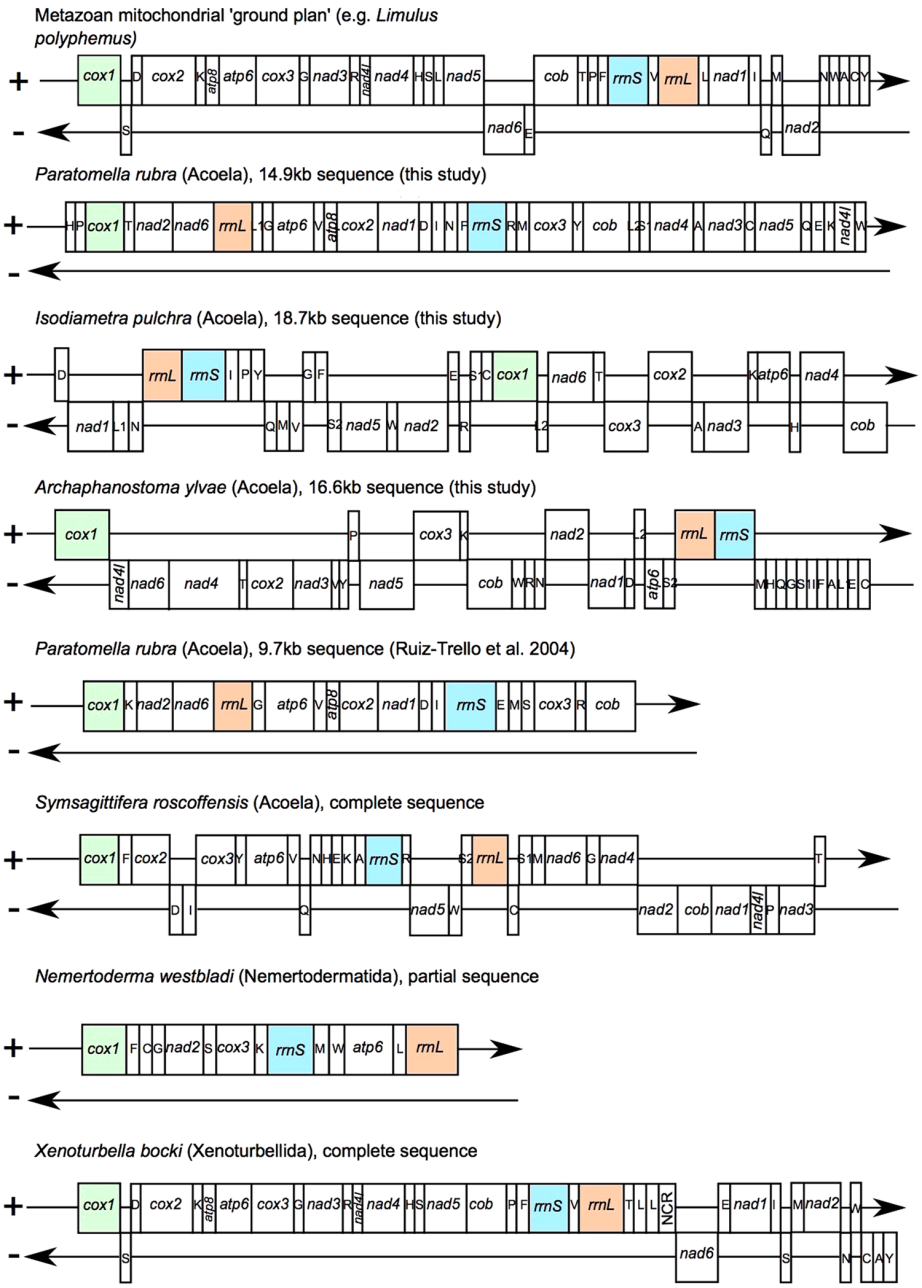
Comparisons of gene orders in the mitochondrial genome sequences resolved for *Paratomella rubra*, *Isodiametra pulchra* and *Archaphanostoma ylvae* compared to a published *P. rubra* fragment; the acoel *Symsagittifera roscoffensis*; the xenoturbellid *Xenoturbella bocki*; the nemertodermatid *Nemertoderma westbladi* and the metazoan mitochondrial ‘ground plan’ gene order, represented by *Limulus polyphemus*. Genes are not drawn to scale. Coloured genes chosen to show ‘anchors’ and divergence from the ground plan gene order in other species.

As was already indicated by the 9.7 kb published partial genome^26^, the gene arrangement we found in *P. rubra* is unique amongst published metazoan mitochondrial genomes. Similarly, neither *I. pulchra* nor *A. ylvae* show any similarity to other published metazoan mitochondrial genomes (Fig. 6). The species analysed in this study share only the small ‘block’ of *nad3-atp6-nad4-cob* (*I. pulchra*) and *cob-nad4-nad3* (*P. rubra*). However, the order is reversed between the two, and the genes are distributed across both strands in *I. pulchra*, so it is unlikely that this represents a feature inherited from a common ancestor. To quantify the number of common gene arrangements between the species in this study and other mitochondrial genomes, protein-coding gene and ribosomal RNA gene order was analysed using CREx^29^ (compared to the acoel *S. roscoffensis*, the xenoturbellid *Xenoturbella bocki*, and the metazoan mitochondrial ‘ground plan’, represented by *L. polyphemus*). Conserved mitochondrial gene ‘blocks’ (that is, a series of genes, regardless of their order within the grouping) were very infrequent between the species. Of the genomes compared, the highest number of common gene blocks was found between *X. bocki* and *P. rubra*. This result was not significant, finding only 16 common intervals out of a possible 150, and confirming the visual observation that gene order between these species is highly variable.

### Phylogenetic analysis, and population differentiation

**Figure 7:**
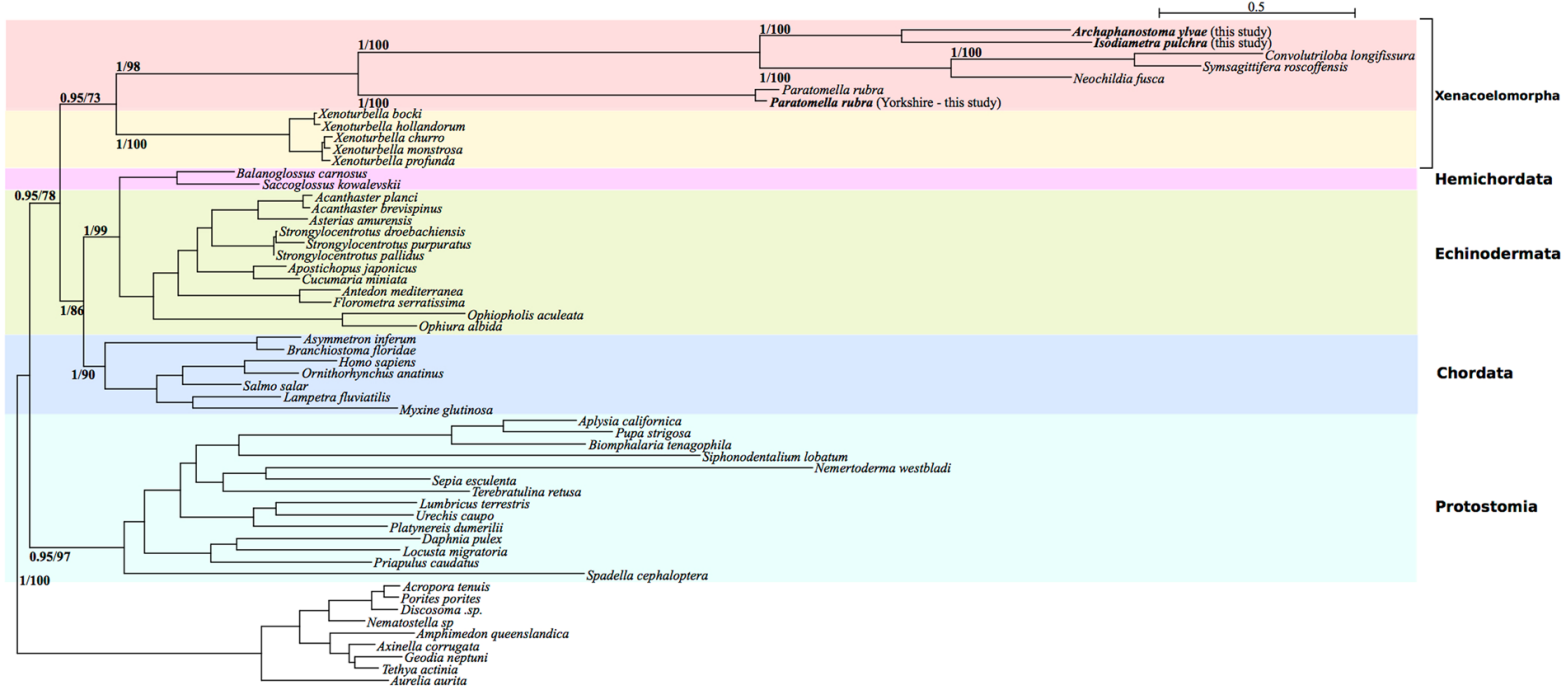
Bayesian (using PhyloBayes^53^) and Maximum Likelihood (using RAxML^51^) phylogenetic analysis of mitochondrial protein-coding genes from the Metazoa, including *P. rubra*, *I. pulchra* and *A. ylvae* with posterior probability and bootstrap support values, respectively, at relevant nodes. Analysis carried out on trimmed alignment. Topology of both trees is identical.

We used our new mitochondrial data from *P. rubra*, *I. pulchra*, and *A. ylvae* to investigate the internal phylogeny of the acoels and to test support for an Acoela-Xenoturbellida affinity. We observed that including the fast-evolving tunicates into our phylogeny leads to a clustering of these species and the acoels in an artificial long-branched clade (Supplementary Figure S1). Removing the tunicates, Bayesian phylogenetic inference was carried out using the protein-coding genes of *P. rubra*, *I. pulchra* and *A. ylvae* on a trimmed concatenated amino acid alignment, including the additional species listed in Supplementary Table S2. The data set reached a MaxDiff of 0.17 after 39,525 trees were sampled across 10 chains discarding the first 400 trees (per chain) as burnin and sampling every 10^th^ tree. In both, this and the maximum likelihood approaches, the protostome/deuterostome split was correctly inferred and Xenacoelomorpha were found splitting off inside Deuterostomia. *P. rubra*, *I. pulchra*, and *A. ylvae* were grouped inside Acoela, as expected (Fig. 7).

We detected the Nemertodermatida species *Nemertoderma westbladi* inside Mollusca and we therefore conclude that the limited data available on Genbank for this species is a contamination.

Having access to the published partial *P. rubra* mitochondrial sequence from a population sampled near Barcelona (Spain) and our own samples from Yorkshire, UK, we could estimate total sequence divergence and compare non-synonymous to synonymous substitutions in eight protein-coding genes found on the Spanish fragment to the same genes from the Yorkshire mitochondrial genome. We found the 9.7 kb sequences to be 82.62% similar at the nucleotide level. The number of substitutions varied between, for example, 23 in the shortest gene alignment (*atp8*; 177 bp), to 161 in *nad2* (972 bp), and 116 in the 1401 bp long *cox1* alignment (Supplementary Table S3). Notably, non-synonymous substitutions appear to be frequent with, for example, 13 in *atp8*, 104 in *nad2*, and 25 in *cox1* (see Supplementary Table S3). Similarity of the *cox1* sequences on the nucleotide level is only 91% over 666 bp, thus higher when compared to species pairs (Supplementary Table S4), but lower than the 95–98% percent threshold used to distinguish species in *cox1* based barcoding^30^.

## Discussion

The 14.9 kb sequence of the *P. rubra* mitochondrial genome determined in our analysis contains the full complement of 37 genes typical of metazoan mitochondrial DNA. Numerous lab-based and computational efforts to close the circular mitochondrial genome were unsuccessful, but the complete gene complement and length of our final sequence indicates that this fragment covers the majority of the *P. rubra* complete mitochondrial genome. The difficulty we encountered in attempting to close the circular mitochondrial sequence may be attributed to the AT-rich, repetitive sequence found at both ends of the fragment, which could have prevented PCR amplification. Similar regions have been shown as problematic in studies of other mitochondrial genomes^31^. As no long stretch of non-coding sequence was found for this species in our study, the missing sequence might represent its mitochondrial transcription control region. Nonetheless, the overall AT content of the *P. rubra* mitochondrial sequence (78.15%) is high even for mtDNA, and greater than the A + T content of the mitochondrial genome of the acoel *S. roscoffensis* (75.3%)^11^ and the published partial *P. rubra* genome (76.4%)^26^.

The validity of the duplicated sequence found in our analysis of the *I. pulchra* mitochondrial genome could not be confirmed by PCR or computational efforts to map short reads to resolve it. Duplications within mitochondrial genomes are not uncommon, and changes to mitochondrial gene order are widely thought to arise as a result of a sequence ‘duplication and deletion’ mechanism^14, 32, 33^. A number of mitochondrial genomes with duplicated sequences have been reported in species with a divergent mitochondrial gene order^33–35^. Given the highly unusual gene order of the *I. pulchra* mitochondrial genome, a genomic duplication such as this could provide evidence for a genomic ‘duplication and deletion’ rearrangement of genes. The rearrangement and separation of protein-coding genes in other mitochondrial genomes has been attributed to long, non-tandem, inverted repeats^34^, and this could be true for *I. pulchra*. Furthermore, very long nematode mitochondrial genomes with variable duplicated regions have been found with a conserved region containing the majority of the protein-coding genes^36^: in *I. pulchra* the protein-coding genes and tRNAs, with the exception of *nad1* and *trnD* and *L1* are found in one long region, outside of the duplicated section. However, long non-coding duplications are frequently adjacent to tRNA*s* or other sequences capable of forming stem-and-loop structures^37^. This is not true for the potential duplicate in *I. pulchra*. Most puzzling, both occurrences of the duplicate are identical, nucleotide-by-nucleotide, and unless the duplication occurred exceptionally recently, it is likely that spontaneous mutations would result in differences between the two copies of the sequence, especially given the elevated mutation rate of mitochondrial genomes. While it is true that the duplicated sequences appear at the start and end point of transcriptome assembly contigs, meaning it is possible that the duplication observed occurred only as a result of a sequencing and assembly error, their existence is nevertheless supported by PCR products which show an identical sequence being adjacent to both *rrnL* and to *cob*.

The 14.9 kb mitochondrial genome of *P. rubra*, the 18.7 kb sequence from *I. pulchra* and the complete 16.6 kb *A. ylvae* mitochondrial genome show no significant organisational similarity to any other published metazoan mitochondrial genome (Fig. 6). Comparison of the 14.9 kb *P. rubra* sequence with the published 9.7 kb *P. rubra* fragment shows an identical protein-coding and ribosomal gene order, but with variation in tRNA order (Fig. 6). tRNAs are reported to show much more frequent gene translocation compared to larger genes^38^, which could account for these discrepancies. This, and the differences on the nucleotide level, including the relatively low level of similarity in the *cox1* barcoding gene might indicate that *P. rubra* collected from Barcelona (Spain)^26^ and our animals from Yorkshire (England) should be regarded as cryptic species and not just divergent populations. Given the large and mostly unresolved diversity in benthic communities^39^, and the marine environment in general^40^, a differentiation into (cryptic) species cannot be seen as surprising.

All species analysed in this study are unique in the orientations and orders of their genes: *P. rubra* has genes exclusively on one strand; *I. pulchra* has an almost-equal distribution of genes across both the plus (18 genes) and minus (17 genes) strand; with *cox1* in a ‘forward’ orientation at the start of the genome, the majority of the protein-coding genes for *A. ylvae* are found on the minus strand. Furthermore, genes in *I. pulchra* and *A. ylvae* are not clustered into groups of ‘gene blocks’ on the same strand, but are found frequently as one or two genes on each strand. The finding of a unique gene order for these species seems to be typical for the acoels: analysis of the complete *S. roscoffensis* mitochondrial genome found no gene order similarity to any other species published to date, suggesting great variability in mitochondrial gene order amongst the acoels^11^.

In addition to an unusual gene order, the mitochondrial genome of *P. rubra* shows frequent overlaps between protein-coding genes and tRNAs. tRNAs have been reported within protein-coding genes in other metazoan mitochondrial genomes^41, 42^, and given that no other location could be predicted for these sequences, this overlap could represent the simultaneous coding for both tRNAs and protein-coding genes. Overlap in coding sequence could be the result of selection to reduce genome size, accompanied by a reduction in non-coding sequence^42^, and truncated tRNAs with incomplete secondary structure^43^, both of which are also found for the *P. rubra* sequence. The opposite is true for the *I. pulchra* and *A. ylvae* sequences. For *I. pulchra*, the sequence we could confidently verify makes the minimal length of the *I. pulchra* mitochondrial genome 18,725 base pairs, and it is likely to be longer in the complete closed circular genome. As has been found for other ‘long’ mitochondrial genomes, this is largely due to a large portion of the genome being non-coding^44^. The lengths of protein-coding genes inferred for *I. pulchra* are similar to those of other acoel species (Table 4), and in addition, two protein-coding genes (*atp8* and *nad4l*) appear to have been lost from the genome, contributing to a reduced proportion of protein-coding gene sequence within the genome. The loss of *atp8* is not unusual, and has been reported in a number of unrelated taxa, as well as *S. roscoffensis* and in our *A. ylvae* mitochondrial genome^11^. The absence of *nad4l* is more unusual, and could be a result of its existence in a portion of the genome that we have been unable to sequence. Although non-coding sequence contributes a relatively large proportion of the *A. ylvae* mitochondrial genome (17.17% compared to 22.72% in the *I. pulchra* sequence), the total genome is not exceptionally long.

**Table 4:** Length of protein–coding genes in acoel mitochondrial genomes. All gene lengths in base pairs.

	*Isodiametra pulchra*	*Paratomella rubra*	*Symsagittifera roscoffensis*	*Archaphanostoma ylvae*
*cox1*	1536	1563	1551	1539
*cox2*	615	663	741	648
*cox3*	798	786	792	786
*nad1*	881	1053	870	870
*nad2*	1053	1014	990	1002
*nad3*	378	390	393	369
*nad4*	1344	1326	1350	1344
*nad4l*	absent	309	270	285
*nad5*	1710	1752	1776	1656
*nad6*	476	462	480	468
*cob*	1134	1083	1161	1086
*atp6*	681	609	702	702
*atp8*	absent	177	absent	absent

The internal phylogeny we resolve for Acoela is in line with that proposed by Jondelius *et al*.^25^. *I. pulchra* and *A. ylvae* group together in the family Isodiametridae. Isodiametridae groups with *S. roscoffensis*, *Neochildia fusca* and *Convolutriloba longifissura*, which are all members of the Convolutidae. *P. rubra* forms a separate branch outside the Convolutidae, representing the Paratomellidae. We interpreted the initial grouping of the acoels and tunicates as a classical example of long branch attraction (LBA) (Supplementary Figure S1). The accelerated substitution rates in mitochondrial DNA are also evidenced by the cryptic divergence we find in *P. rubra*, and may well lead to LBA in phylogenies derived from mitochondrial protein-coding genes, owing to the clustering of rapidly evolving lineages. This is of particular relevance for acoel species, which already demonstrate a very rapid rate of nucleotide substitution compared to other metazoans, leaving them vulnerable to LBA. Excluding the urochordates, we do retrieve Xenacoelomorpha as a branch of the deuterostomes, as expected (Fig. 7).

The mitochondrial genome sequences we analysed for the acoel species *P. rubra*, *I. pulchra* and *A. ylvae* have very divergent gene orders compared to other metazoan species. Furthermore, these species have very different mitochondrial features: a large amount of genomic overlap in *P. rubra*, and a lot of non-coding sequence in *I. pulchra* and *A. ylvae*. It is also possible that the mitochondrial genome of *I. pulchra* has a non-tandem inverted duplication - which could provide a mechanism for gene order variation - but this could not be confirmed by lab or computational based methods. Although limited to four species, the uniqueness of acoel mitochondrial genomes analysed so far^11, 26^ means that gene order and other mitochondrial genome features may not be phylogenetically informative for this order, although further mitochondrial genomes from other members of the Acoela would no doubt aid in this comparative analysis. Similarly, the cryptic divergence found between *P. rubra* samples from Yorkshire and Barcelona illustrate the usefulness of studying mitochondrial genomes to understand hidden species diversity. Our data clearly emphasise the still problematic placing of Xenacoelomorpha, with the clade firmly placed inside deuterostomes, but LBA drawing the acoels towards the outgroup when the tunicates were included. In summary, more data from genomes of early branching taxa are needed to resolve phylogenetic and biological questions.

## Methods

### Specimen collection, DNA extraction and PCR

Live *Paratomella rubra* specimens were isolated from sand samples collected from Filey, North Yorkshire and were immediately frozen and stored at −70 °C following identification. Specimens of *Isodiametra pulchra* were cultured in petri dishes with nutrient-enriched f/2 sea water and fed *ad libitum* on *Nitzschia curvilineata* diatoms. DNA was extracted from live specimens of *I. pulchra* and frozen specimens of *P. rubra* using the QIAamp DNA Micro Kit (Qiagen: Product No. 56304) with the manufacturer recommended protocol.

All PCRs were done using the GeneAmp PCR System 2700 (Applied Bioscience). PCRs were carried out using the Expand Long-Range PCR Kit (Roche Applied Sciences: Product No. 11681834001), following manufacturer recommendations for 50 μl reaction set-up. General cycling protocol was: 92 °C for 2 min; 15 cycles of: 92 °C for 10 sec, 57 °C for 15 sec, 68 °C at initial elongation time (approximated as 1 min per 1000 base pairs to be amplified); 2 cycles each of: 92 °C for 10 sec, 57 °C for 15 sec, 68 °C at 40 sec longer than initial elongation time, repeated at increasing 40 sec intervals for a further 14 cycles; a final elongation stage at 68 °C for 7 min and a 4 °C ‘hold’ stage. Where PCRs were not successful using this protocol, they were repeated using the Q5 High-Fidelity PCR Kit (New England Biolabs: Product No. E0555L), following manufacturer recommendations for a 25 μl reaction. Cycling protocol was: 92 °C for 2 min; 40 cycles of: 92 °C for 10 sec, 58 °C for 15 sec, 68 °C at initial elongation time (approximated as 1 min per 1000 base pairs to be amplified); a final elongation stage at 68 °C for 7 min. Amplified products were visualised on ethidium-bromide stained TAE 0.8% gels. Bands of expected size were purified using the High Pure PCR Product Purification Kit (Roche Applied Sciences: Product No. 11732668001) and sent for sequencing by Source BioScience Life Sciences. Only amplifications that resulted in one clear band on the TAE 0.8% agarose gel were sequenced.

Three fragments of sequence from the mitochondrial genome of *P. rubra*, of size ~5.8 kb, ~4 kb, and ~1.2 kb, were generated from our gDNA assembly. Fragments were verified using a translated nucleotide query blast with invertebrate codon usage (blastx NCBI), and their orientation determined by gene annotation in comparison to the published 9.7 kb section of the *P. rubra* mitochondrial genome^26^. Primers were designed in conserved gene regions to:

(1) Amplify across the ‘N-stretches’ present in the 5.8 kb and 4 kb fragments (8 and 9 N-stretches respectively, all of arbitrary length 50 base pairs).

(2) Cover the whole 1.2 kb fragment, with the aim of resolving the two frameshift mutations within the assembled sequence.

(3) Close the circular mitochondrial genome, by joining the 5.8 kb fragment to the 1.2 kb fragment; the 1.2 kb fragment to the 4 kb fragment; and the 4 kb fragment to the 5.8 kb fragment (see Supplementary Figure S5). Amplification of the fragments joining the 1.2 kb fragment to the 4 kb fragment and to close the mitochondrial genome using standard PCR cycling were unsuccessful. These were repeated using a touchdown protocol with Expand Long-Range polymerase. Annealing temperature was set at 65 °C with decreasing 2 °C intervals every 2 cycles down to 49 °C. Initial elongation time was calculated as before, increasing 30 sec every two cycles of the touchdown, with a final 6 cycles at 49 °C. This successfully amplified the region joining the 1.2 kb fragment to the 4 kb fragment, but we could not close the circular genome. Design of three new forward and reverse primers, tried in all combinations and using variable PCR parameters were unsuccessful in closing the mitochondrial genome. Additional RNA-Seq and DNA genomic sequencing data corroborated the stretches of sequence at either end of the mitochondrial genome but did not aid in closing the circle.

Three mitochondrial contigs of size ~13 kb, ~3.5 kb and ~1.3 kb were identified from *I. pulchra* Trinity transcriptome assembly from total RNA sequencing. A further contig of ~19 kb was also identified, covering the entire ~1.3 and 13 kb regions, and ~2.4 kb of the 3.5 kb sequence. Fragments were verified using blastx, NCBI, as outlined for *P. rubra*, and approximations for the location of protein-coding genes and tRNAs determined using the MITOS mitochondrial genome annotation server^45^ (http://mitos.bioinf.uni-leipzig.de/help.py). Primers were designed to span the 13 kb contig in two ~5 kb sections, and to join the 13 kb contig to the 3.5 kb contig in both directions, to close the mitochondrial genome and check the validity of the duplicated region (Fig. 3). RNA-Seq data for *I. pulchra* were mapped to the long transcriptome assembly contigs and PCR sequencing results using NextGenMap^46^, and visualised using Tablet^47^.

We accidentally co-sequenced *A. ylvae* at very low coverage as part of a *P. rubra* genome sequencing experiment. From an initial paired end assembly of Illumina HiSeq data with the CLC assembly cell software (v.5.0) we extracted the full mitochondrial circle of *A. ylvae* in a single contig identified with BLAST. This was then annotated using MITOS and manual refinement as described above.

### Genome annotation

For *P. rubra*, all sequenced fragments were aligned against the initial scaffold of the 9.7 kb published sequence^26^; the 5.8 kb, 4 kb and 1.2 kb genome assembly sequences; and an additional long genome assembly fragment of length 14,954 (see Supplementary Figure S5). All contigs and PCR sequencing results were similarly aligned for *I. pulchra*, but without a reference sequence (see Fig. 3). Alignments were visualised using Mesquite (http://mesquiteproject.org) with invertebrate mitochondrial translated amino acid state colour coding. Where ambiguity remained between PCR sequencing results and genome or transcriptome assembly fragments, the genome or transcriptome assembly nucleotide sequence was used to establish a final ‘consensus’ sequence for each mitochondrial genome. This was of particular relevance for repetitive AT regions – for example, within *P.rubra nad1*, for which PCR sequencing results were inconclusive. In the case of *I. pulchra*, where the validity of the duplicated sections could not be confidently determined, we resolved a consensus sequence of 18, 725 base pairs (Fig. 3).

The region for each protein-coding mitochondrial gene (*nad1*–*6, nad4l, cox1*–*3, cob, atp6 and atp8*) in the *P. rubra*, *I. pulchra* and *A. ylvae* sequences were compared against published mitochondrial genomes using a translated nucleotide query (blastx, NCBI) with NCBI translation table number 5 ‘invertebrate mitochondrial’. Published genes from the mitochondrial genomes of the acoels *Symsagittifera roscoffensis* and *P.rubra* were downloaded from the NCBI Nucleotide database and aligned to the new consensus gene sequences of both *P. rubra*, *I. pulchra* and *A. ylvae* to verify the location of protein-coding and ribosomal RNA-encoding genes. The 5′ end of protein-coding genes were inferred to start from the first in-frame start codon (ATN, GTG, TTG, or GTT), even if this appeared to overlap with the preceding gene. Similarly, the terminal of protein-coding genes was inferred to be the first in-frame stop codon (TAA, TAG, or TGA). If no stop codon was present, a truncated stop-codon (T– or TA-) prior to the beginning of the next gene was assumed to be the termination codon, completed by post-transcription polyadenlylation. tRNA sequences and putative secondary structures were identified using the Mitfi program within MITOS.

### Sequence alignment, phylogenetic analysis, and evolutionary rates

Phylogenetic analysis was performed using a concatenated amino acid alignment of all thirteen protein-coding genes for *P. rubra*, and all eleven protein-coding genes present in *I. pulchra*. Nucleotide sequences from all three acoel taxa and an additional set of species comprising 54 metazoans, taken from a range of published metazoan mitochondrial genomes representing deuterostomes, protostomes, cnidarians, and two species of poriferans as an outgroup (Supplementary Table S2) were aligned using TranslatorX (http://www.translatorx.co.uk/) independently for all genes with the appropriate mitochondrial genetic code set for each taxon included, using ClustalOmega^48^ for amino acid alignment (Supplementary Table S2). Protein alignments were reduced to the most informative residues using trimAl v.1.4.rev15^49^ with standard settings. Regions showing ambiguity in alignment were excluded, so that only blocks of well-aligned sequence were included for analysis.

We initially re-constructed neighbour nets in SplitsTrees v.4^50^ to screen our dataset for potentially non-treelike patterns, which could impede our phylogenetic analysis. Subsequently, we used RAxML^51^ v. 8.2.9 to infer maximum likelihood phylogenies from the original and the reduced alignments under the MTZOA model^52^. Bootstrapping was conducted employing the ‘autoMRE’ option in RAxML and the trees visualised with figtree v.1.4 (http://tree.bio.ed.ac.uk/software/figtree). We carried out Bayesian inference on the same trimmed alignment with PhyloBayes v.4.1^53^ under the MTZOA model. We ran 10 chains in parallel and stopped the tree search at ~3950 trees per chain, with a maximum difference of 0.17, when discarding 400 trees as burnin and sampling every 10^th^ tree per chain.

We used the Geneious software (v.8) to calculate sequence differences between a 9.7 kb section of the *P. rubra* mitochondrial genome originating from worms sampled in Filey, Yorkshire (UK) and Barcelona (Spain)^26^. For eight protein-coding genes found on this section we used ParaAT (v2.0)^54^ to calculate translation alignments and the KaKs calculator (v1.2)^55^ to access substitution rates. Also using Geneious we calculated a difference matrix for the *cox1* barcoding gene of the two *P. rubra* populations in comparison to acoel coI sequences retrieved from Genbank.

## Electronic supplementary material

Supplementary Information

Supplementary Dataset File S4
