# Supplementary material for: The mitochondrial genomes of the acoelomorph worms Paratomella rubra, Isodiametra pulchra and Archaphanostoma ylvae
Source: Sci Rep. 2017 May 12;7:1847. doi: 10.1038/s41598-017-01608-4 (PMC5431833; doi:10.1038/s41598-017-01608-4)
Supplement: Supplementary file 1 — Supplementary Information [file 41598_2017_1608_MOESM1_ESM.pdf]

Supplementary Information: The mitochondrial genomes of the acoelomorph worms *Paratomella rubra, Isodiametra pulchra* and *Archaphanostoma ylvae*

Helen E Robertson, François Lapraz, Bernhard Egger, Maximilian J Telford and Philipp H. Schiffer

Supplementary Figure S1: Bayesian (using PhyloBayes[53]) phylogenetic analysis of mitochondrial protein-coding genes from the Metazoa, including initial date from *P. rubra* and *I. pulchra*, with posterior probabilities relevant nodes. Analysis carried out on trimmed alignment. (See Supplementary Figure S1 PDF)

Supplementary Table S2: Accession numbers (NCBI) for taxa used in study

Classification	Species	Accession Number
**Acoela**	*Paratomella rubra*	AY228758
	Paratomella rubra (this study)	submitted
	Symsagittifera roscoffensis	NC_014578.1
	Isodiametra pulchra (this study)	submitted
	Convolutriloba longifissura	Trace Archive<br>Library_id=CT_MM1
	Neochildia fusca	Trace Archive<br>Library_id=NF_MM1
**Annelida**	*Platynereis dumerilii*	AF178678
	Urechis caupo	NC_006379
	Lumbricus terrestris	NC_001673.1
**Arthropoda**	*Locusta migratoria*	NC_001712
	Daphnia pulex	NC_000844
**Brachiopoda**	*Terebratulina retusa*	NC_000941.1
**Chaetognatha**	*Spadella cephaloptera*	NC_006386.1
**Chordata**	*Salmo salar*	LC012541.1
	Homo sapiens	NC_001807
	Lampetra fluviatilis	NC_001131
	Ornithorhynchus anatinus	NC_000891.1
	Branchiostoma floridae	NC_000834.1
	Asymmetron inferum	NC_009774.1
	Myxine glutinosa	NC_002639.1
**Cnidaria**	*Acropora tenuis*	NC_003522.1
	Porites porites	NC_008166
	Discosoma sp	NC_008071.1
	Nematostella sp	NC_008164
	Aurelia aurita	NC_008446.1
**Echinodermata**	*Antedon mediterranea*	NC_010692.1
	Florometra serratissima	NC_001878.1
	Strongylocentrotus pallidus	NC_009941.1
	Strongylocentrotus droebachiensis	NC_009940.1
	Strongylocentrotus purpuratus	X12631.1
	Acanthaster brevispinus	NC_007789.1
	Acanthaster planci	NC_007788.1
	Asterias amurensis	NC_006665.1
	Apostichopus japonicus	NC_012616.1
	Cucumaria miniata	NC_005929
	Ophiura albida	NC_010691.1
	Ophiopholis aculeata	NC_005334.1
**Hemichordata**	*Balanoglossus carnosus*	NC_001887.1
	Saccoglossus kowalevskii	NC_007438
**Mollusca**	*Sepia esculenta*	NC_009690.1
	Aplysia californica	NC_005827.1
	Pupa strigosa	NC_002176
	Biomphalaria tenagophila	NC_010220.1
	Siphonodentalium lobatum	NC_005840.1
**Nemertodermatida**	*Nemertoderma westbladi*	AY228757.1
**Porifera**	*Geodia neptuni*	NC_006990.1
	Axinella corrugata	NC_006894.1
	Tethya actinia	NC_006991.1
	Amphimedon queenslandica	NC_008944
**Priapulida**	*Priapulus caudatus*	DQ463747
**Urochordata**	*Ciona intestinalis*	NC_004447.2
	Doliolum nationalis	AB176541.1
**Xenoturbellida**	*Xenoturbella bocki*	NC_008556.1
	Xenoturbella hollandorum	NC_029218.1
	Xenoturbella monstrosa	NC_029219.1
	Xenoturbella churro	NC_029217.1
	Xenoturbella profunda	NC_029220.1

Supplementary Table S3: Substitution pattern in eight genes found on the published *Paratomella rubra* mitochondrial genome (sampled from Barcelona, Spain) in comparison to our samples from Yorkshire, UK.

Sequence	Length	S-Sites	N-Sites	Substitutions	S-Substitutions	N-Substitutions
atp6	594	90.8602	503.14	70	40.9815	29.0185
atp8	177	15.4355	161.565	23	10	13
cob	795	113.09	681.91	89	63.9542	25.0458
cox1	1401	158.705	1242.3	116	90.904	25.096
cox2	660	116.441	543.559	81	50.9427	30.0573
cox3	780	99.2387	680.761	87	60.5296	26.4704
nad1	930	95.8574	834.143	103	63.3614	39.6386
nad2	972	156.176	815.824	161	56.7858	104.214
nad6	330	44.3145	285.685	52	31.121	20.879

Supplementary Dataset File S4: Sequence identity over 666 base pairs of *cox1* (See Spreadsheet Supplementary Dataset File S4)

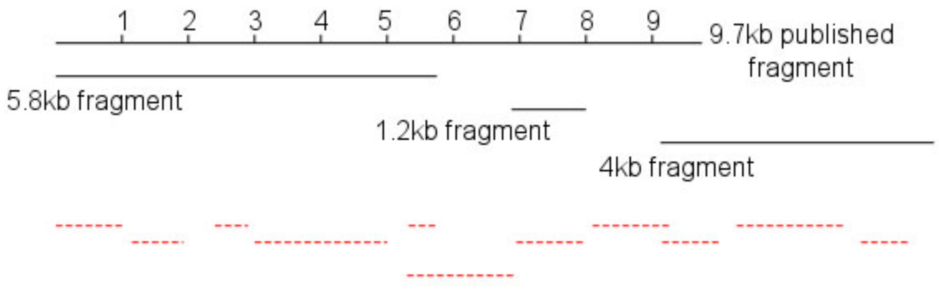

Supplementary Figure S5: Schematic of *Paratomella rubra* genome assembly fragments used as an initial starting point for mitochondrial genome inference. PCR sequencing results bridging or covering parts of these fragments indicated by red dashed lines.

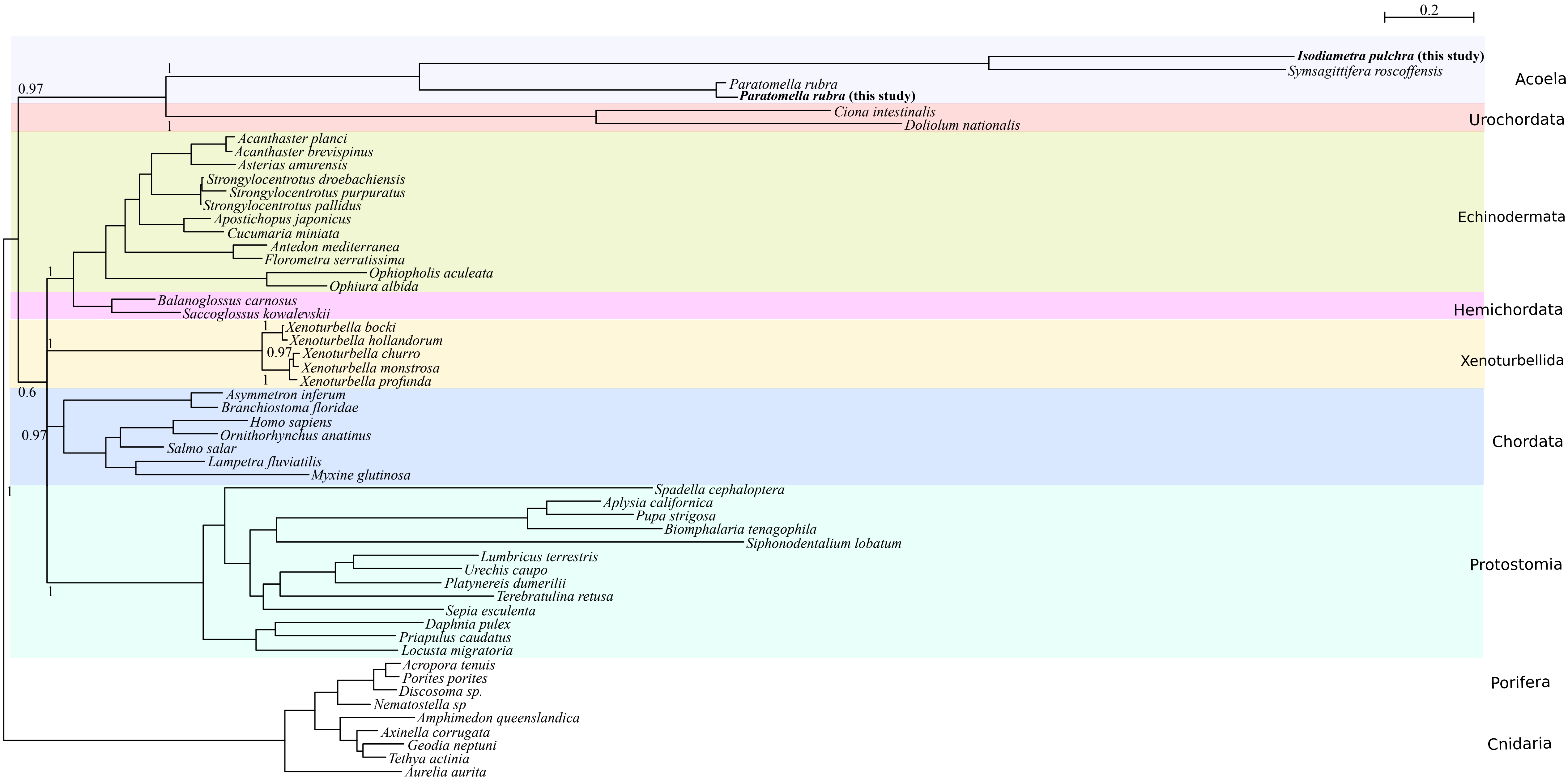
0.2
Isodiametra pulchra (this study)
Symsagittifera roscoffensis
Paratomella rubra
Paratomella rubra (this study)
Ciona intestinalis
Doliolum nationalis
Acanthaster planci
Acanthaster brevispinus
Asterias amurensis
Strongylocentrotus droebachiensis
Strongylocentrotus purpuratus
Strongylocentrotus pallidus
Apostichopus japonicus
Cucumaria miniata
Antedon mediterranea
Florometra serratissima
Ophiopholis aculeata
Ophiura albida
Balanoglossus carnosus
Saccoglossus kowalevskii
Xenoturbella bocki
Xenoturbella hollandorum
Xenoturbella churro
Xenoturbella monstrosa
Xenoturbella profunda
Asymmetron inferum
Branchiostoma floridae
Homo sapiens
Ornithorhynchus anatinus
Salmo salar
Lampetra fluviatilis
Myxine glutinosa
Spadella cephaloptera
Aplysia californica
Pupa strigosa
Biomphalaria tenagophila
Siphonodentalium lobatum
Lumbricus terrestris
Urechis caupo
Platynereis dumerilii
Terebratulina retusa
Sepia esculenta
Daphnia pulex
Priapulus caudatus
Locusta migratoria
Acropora tenuis
Porites porites
Discosoma sp.
Nematostella sp
Amphimedon queenslandica
Axinella corrugata
Geodia neptuni
Tethya actinia
Aurelia aurita
Acoela
Urochordata
Echinodermata
Hemichordata
Xenoturbellida
Chordata
Protostomia
Porifera
Cnidaria
